# Break to Build: Isothermal Assembly of Nucleic Acid Nanoparticles (NANPs) *via* Enzymatic Degradation

**DOI:** 10.1021/acs.bioconjchem.3c00167

**Published:** 2023-06-09

**Authors:** Damian Beasock, Anh Ha, Justin Halman, Martin Panigaj, Jian Wang, Nikolay V. Dokholyan, Kirill A. Afonin

**Affiliations:** † Nanoscale Science Program, Department of Chemistry, University of North Carolina at Charlotte, Charlotte, North Carolina 28223, United States; ‡ Department of Pharmacology, Department of Biochemistry & Molecular Biology, Penn State College of Medicine, Hershey, Pennsylvania 17033, United States; § Department of Biochemistry & Molecular Biology, Department of Biochemistry & Molecular Biology, Penn State College of Medicine, Hershey, Pennsylvania 17033, United States

## Abstract

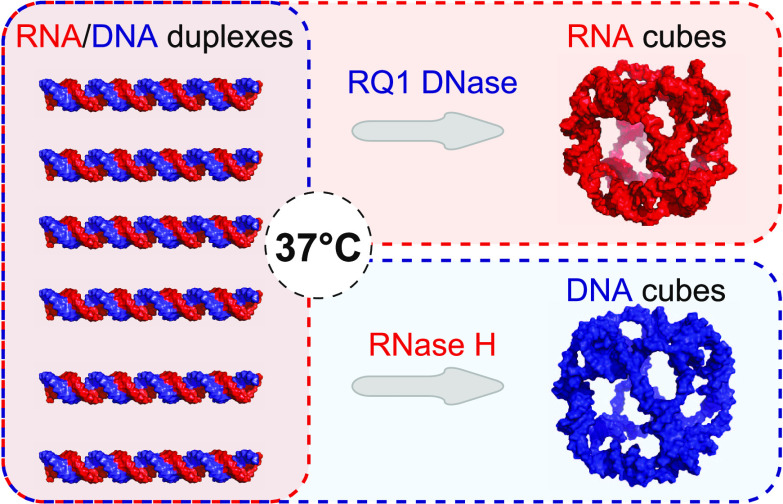

The intrinsic properties of RNA and DNA biopolymers emphasized by engineered nucleic acid nanoparticles (NANPs) offer accelerated development of next-generation therapies. The rational design of NANPs facilitates programmable architectures intended for regulated molecular and cellular interactions. The conventional bottom-up assembly of NANPs relies on the thermal annealing of individual strands. Here, we introduce a concept of nuclease-driven production of NANPs where selective digestion of functionally inert structures leads to isothermal self-assembly of liberated constituents. The working principles, morphological changes, assembly kinetics, and the retention of structural integrity for system components subjected to anhydrous processing and storage are assessed. We show that the assembly of precursors into a single structure improves stoichiometry and enhances the functionality of nuclease-driven products. Furthermore, the experiments with immune reporting cell lines show that the developed protocols retain the immunostimulatory functionality of tested NANPs. The presented approach enables exploitation of the advantages of conditionally produced NANPs and demonstrates that NANPs’ stability, immunorecognition, and assembly can be regulated to allow for a more robust functional system.

## Introduction

Self-assembly is a ubiquitous natural process wherein molecules spontaneously organize themselves in higher-order structures based on noncovalent interactions.^1^ This phenomenon encompasses a broad spectrum of assembly examples involving many molecular structures including nucleic acids, proteins, and lipids making their self-assembled structures indispensable for life.^2^ Many assemblies, such as the cytoskeleton, are in a continual dynamic process of regulated assembly and disassembly. Although the molecular rearrangement of this system is spontaneous, cells maintain substantial control over the molecular associations.^3^ The working principles of these active structures have already inspired the development of responsive, shape-switching protein and nucleic acid-based nanosystems.^4−6^

The self-assembling nucleic acid nanoparticles (NANPs) are an advanced class of biomaterials comprised of programmable combinations of structural and functional RNA and DNA motifs embedded within the same unique architectures. Assembled NANPs often gain new physicochemical properties that can be quite distinct from their original constituents but still retain the functional roles of nucleic acids.^7^ Significantly, the immunomodulatory properties of NANPs also differ from the unassembled monomers and can be rationally designed to illicit expected immune responses that depend on NANPs’ architectures and routes of delivery.^8−10^

**Scheme 1:**
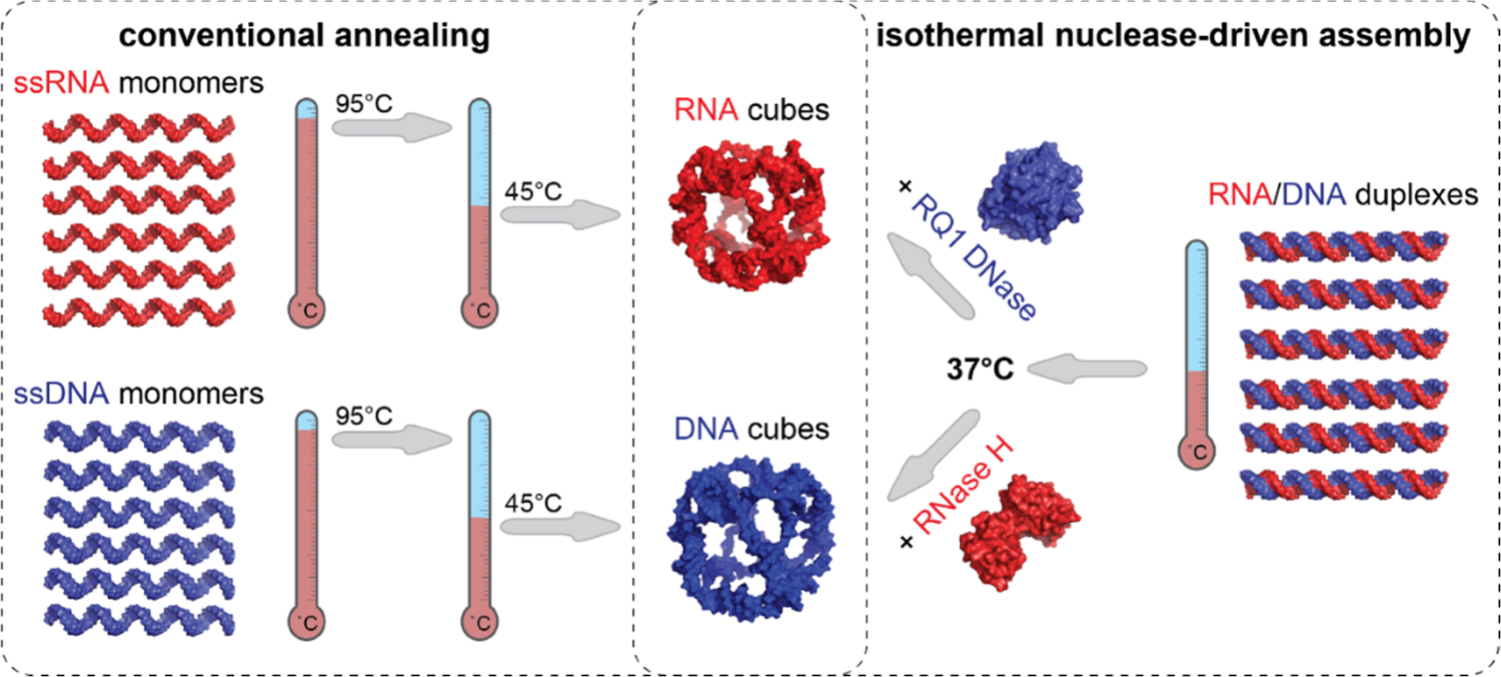
Comparison of Conventional One-Pot Annealing Protocols with One-Pot Isothermal Nuclease-Driven Assembly of Nucleic Acid Nanoparticles

The conventional bottom-up assembly of NANPs is typically a multistep process that requires combining individually synthesized and characterized constituent strands, followed by different annealing protocols that are specific to individual NANPs’ design and composition (Scheme 1, left panel).^11^ The correctly assembled 3D structures of NANPs play a prominent role in the spatial organization of functional moieties and define their immunomodulation.^12,13^ While widely used, the high level of technicality of the existing assembly protocols and the lack of control over the triggered formation of NANPs at isothermal conditions preclude their broader biomedical applications.

We report a novel “break to build” strategy for NANP self-assembly that relies on a single incubation step with substrate-specific nucleases, either RQ1 DNase or RNase H, to selectively digest the intended constituents of the precursor structures (Scheme 1, right panel). The cleavage of selected strands within the inert RNA/DNA precursors releases the complementary sequences that adopt the next most thermodynamically favorable conformations and result in a particular NANP formation. Notably, cleavage and self-assembly occur simultaneously and under the same conditions at 37 °C, the normal temperature of the human body and a benchmark for all therapeutics.^14,15^ In this work, as a model system, we selected NANPs with cube connectivity due to significant immunostimulatory differences between DNA and RNA cubes reported in previous studies.^6,16^ Our technique circumvents multiple incubation steps and the use of advanced equipment while providing an advantage for the upscaled production of NANPs following established user-friendly procedures. This proof-of-concept study offers the potential to originate a new branch of nucleic acid nanotechnology where enzymes govern the selective degradation of delivered materials and initiate the assembly of expected functional NANPs within cells.

## Results

### Dynamics of Nuclease-Driven Assembly of Cube Nanoparticles

**Figure 1:**
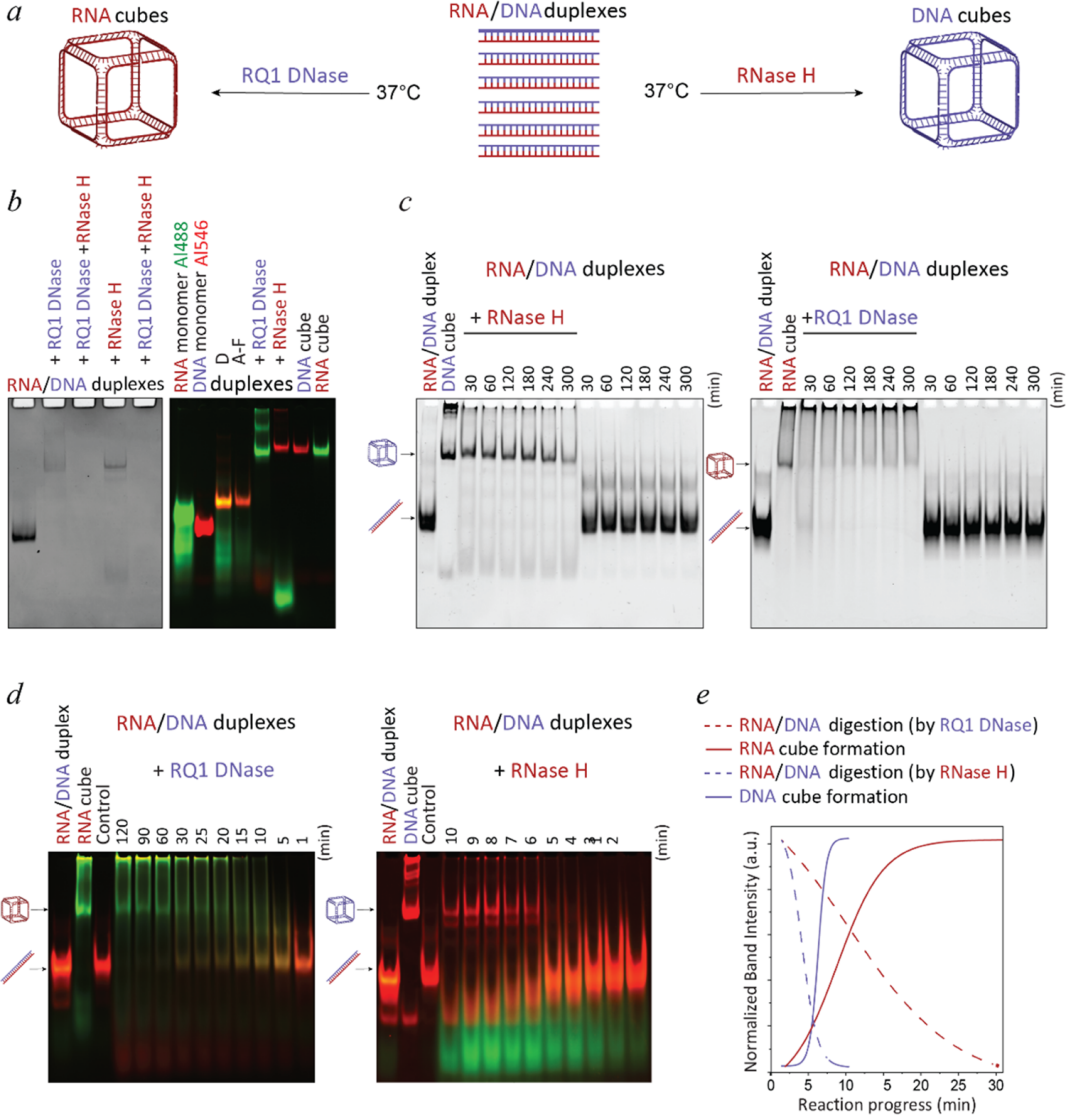
Nuclease-driven assembly of RNA and DNA NANPs. (a) Scheme of NANPs’ assembly via enzymatic degradation by the “break to build” approach. (b) Confirmation of RNA and DNA cube formations triggered by nuclease treatment via ethidium bromide total staining (left) and fluorescent labeling (right). (c) Time course-dependent nuclease-driven assembly of cube NANPs. Hybrid duplexes are treated with nucleases at 37 °C for 5 h and compared to untreated mixtures of hybrids. In highlighted time points, assemblies are moved to 4 °C and incubated until 5 h. (d) Short-term assembly dynamics of fluorescently labeled RNA or DNA strands. (e) Kinetics curves calculated based on the analysis of gel results (mean of *n* = 3).

The addition of RQ1 DNase to hybrid RNA/DNA duplexes leads to the assembly of RNA cubes (Figure 1a), and subsequent treatment of RNA cubes with RNase degrades all RNA assemblies, as shown by non-denaturing polyacrylamide gel electrophoresis (native-PAGE) experiments. Similarly, RNase H releases ssDNA monomers from hybrid duplexes, creating DNA cubes that can then be degraded by RQ1 DNase (Figure 1b). Conjugation of individual monomers with Alexa 488 and Alexa 546 fluorophores allows for the tracking of shift in gel migration and color change after individual strands bind to their complementary counterparts. Conventional assembly of NANPs is used as a standard for comparison to the products of nuclease-driven assembly reactions. In both cases, cubes formed by nuclease-driven assemblies have the same molecular weight as controls. However, it is revealed that depending on nucleases, the RNA and DNA cubes assemble at different rates.

The DNA cubes are formed within the minutes after RNase H addition (Figures 1c and S1a). Interestingly, the prolonged incubation of up to ∼5 h at 37 °C does not improve the DNA cubes’ assembly efficiencies (Supporting Information (SI) Figure S1b), suggesting a relatively quick digestion of hybrid duplexes by RNase H and reaching the plateau phase of assembly. The extended storage at 4 °C maintains the high yields of DNA cube assemblies (Figure S2). When the incubation with RNase H is carried out at 4 °C, the decreased digestion rates slow down the formation of DNA cubes (Figure S1c). Importantly, no cubes are observed during the extended incubation of RNA/DNA duplexes without nucleases. It should also be noted that DNA cubes show less aggregation remaining in the loading wells compared to cubes prepared by the conventional assembly protocol. This could be attributed to the continuous release of individual monomers that lowers their concentration during the assembly that occurs at the DNA cubes’ melting temperature.^6^ Initial assessment of assembly dynamics shows that RNase H-driven assembly of DNA cubes starts after ∼5 min, and all monomers are assembled in cubes after ∼10 min (Figures S1a and S2). Upon closer analysis using 1 min intervals, it is revealed that all DNA cubes are formed at ∼6 min, and the amount does not increase significantly thereafter (Figure 1c). The assembly yields of DNA cubes are not affected by extended incubations at 4 °C (Figure S2).

RQ1 DNase-driven assembly of RNA cubes requires a significantly longer time with some RNA cubes only appearing after ∼30 min of incubation. However, the presence of RNA/DNA hybrid duplexes can still be observed for up to 1 h (Figures 1d, S3a). Compared with the control assembly, the proportion of RNA materials trapped in the native-PAGE gel wells is higher. There is also no observed dependence of RNA cube assembly yields on storage at 4 °C (Figure S3b). Interestingly, RQ1 DNase-driven assembly of RNA cubes can only be achieved at 37 °C with no cube formation observed after incubation at 4 °C for 5 h (Figure S3c,d).

Further comparative analysis of nuclease-driven assembly kinetics shows that RQ1 DNase is significantly slower in degrading hybrid duplexes (∼30 min) and releasing ssRNA strands, while RNase H digests its RNA substrates in RNA/DNA hybrids at much faster rates (∼10 min) (Figure 1d,e).

**Figure 2:**
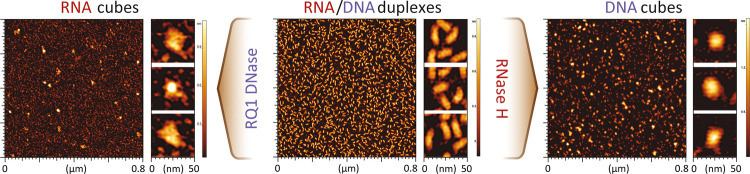
AFM images of RNA and DNA cubes assembled during the nuclease cleavage of RNA/DNA hybrid duplexes.

In addition to electrophoretic mobility shift assays, the homogeneity of resulting NANPs and the nuclease-driven changes in morphology from RNA/DNA duplexes to cubes are confirmed by atomic force microscopy (AFM) (Figure 2). While the dimensionalities of RNA and DNA cube structures are too small to be characterized in detail by AFM, their images are consistent with previous studies^6,13,17^ and the short linear RNA/DNA hybrids are no longer present, indicating that the nucleic acid materials are successfully restructured after the nuclease digestion.

### Storage and Thermal Stability of Precursor Duplexes

Currently, the standard storage and transportation of NANPs in solution require a cold chain that is costly and, under some circumstances, challenging. Our previous study shows that various NANPs retain their structures differently when subjected to dehydration methods and subsequent storage conditions.^18^ SpeedVac is one of the simplest available laboratory techniques with the most promise for future upscaling and cost-effectiveness when addressing storage possibilities outside the cold chain. However, when compared to other dehydration techniques (e.g., lyophilization and light-assisted drying), SpeedVac was shown to have the most negative impact on the structure and recovery of RNA and DNA cubes.

The advantage of the presented “break to build” approach is that from one composition of simple precursor structures, two different NANPs can be produced. Also, RNA/DNA hybrid duplexes are expected to remain intact when dried by any dehydration method available. Therefore, we investigate how the SpeedVac dehydration affects the storage and nuclease-driven assembly of NANPs (Figure S4a). Possible harsh conditions during shipping and storage outside the cold chain are simulated by storing the representative samples at +50 °C for 24 h. Unlike DNA cubes, the integrity of RNA cubes, incubated at +50 °C both in solution and after dried by SpeedVac, is lost and cannot be recovered after rehydration. As expected, RNA/DNA hybrid duplexes resist elevated temperatures both in dehydrated conditions and in solution and can be readily converted to either RNA or DNA cubes after treatment with corresponding nucleases (Figure S4b).

### Assembly of Precursor Duplexes to Long RNA/DNA Hybrid Fibers

**Figure 3:**
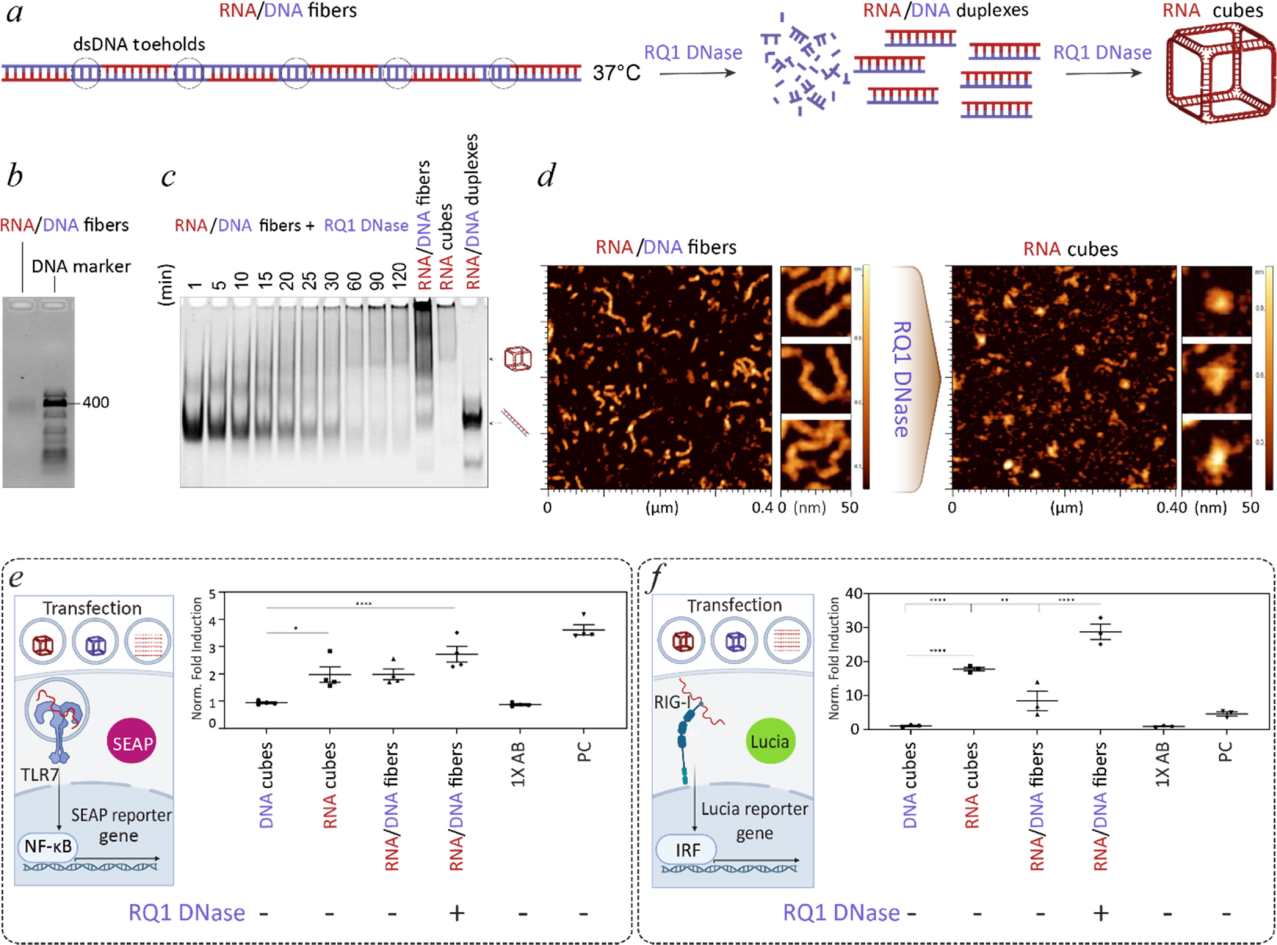
RQ1 DNase-driven assembly of RNA cubes from RNA/DNA fibers and their immunostimulation assessed in reporter cell lines. (a) Schematics of DNase-driven transition for RNA/DNA fibers to RNA cubes. The proposed mechanism assumes two-steps: degradation of dsDNA toeholds, followed by digestion of hybrid duplexes and cubes assembly. (b) Confirmation of RNA/DNA fiber assembly by the agarose gel. (c) Stepwise degradation of RNA/DNA fibers by RQ1 DNase and assembly of RNA cubes confirmed by native-PAGE. (d) AFM images of RNA/DNA fibers and resulting RNA cubes. (e) Scheme of TLR7 activation with subsequent expression and secretion of SEAP to cell media. Normalized fold induction of TLR7 by individual treatments to untreated cells (*n* = 4, ± SEM). (f) Scheme of RIG-I activation with subsequent expression of Lucia luciferase that is secreted to cell media and normalized fold induction of RIG-I by individual treatments to untreated cells (*n* = 3, ± SEM). (e, f) Statistical analysis by ordinary one-way ANOVA (*p* < 0.05).

To improve stoichiometry of individual NANP components and enhance their equimolar assembly, six complementary to RNA monomers DNA strands with five 15-mer toeholds are designed to assemble RNA/DNA duplexes into the hybrid fibers (Figure 3a). Assembly of DNA holding strands with RNA cube components results in 396 bp long fibers, as confirmed by agarose electrophoresis (Figure 3b). The addition of RQ1 DNase leads to rapid digestion of dsDNA toeholds and liberation of RNA/DNA hybrids (Figure 3c). In the following step, RQ1 DNase degrades DNA strands in RNA/DNA hybrids and RNA cubes assemble, in agreement with previous results. The nuclease-driven transition of linear RNA/DNA fibers to 3D RNA cubes can be additionally confirmed by AFM (Figure 3d).

### Immunostimulatory Properties of Nuclease-Driven Assemblies

Various NANPs have been tested to induce the activation of relevant pattern recognition receptors (PRR). We examined the immunostimulatory properties of our assemblies in reporter cell lines that express TLR7 (Toll-like receptor 7) or RIG-I (retinoic acid-inducible gene I). TLR7 is an endosomal receptor sensing ssRNAs and short dsRNAs in NANP structures, while RIG-I is a cytosolic receptor that recognizes RNA NANPs with triphosphate on the 5′ ends.^13,19−22^ Naturally, cytokine production results from signaling pathways triggered through either PRR. Stimulation of TLR7 and RIG-I in engineered reporter cell lines activates expression and secretion of SEAP (secreted embryonic alkaline phosphatase) and luciferase, respectively (Figure 3e,f). Consistently with previous reports,^19^ all transfections containing RNA cube structures show significantly higher activation of TLR7 when compared to DNA analogues (Figures 3e, S6a). For HEK-Lucia RIG-I reporter cells, none of the transfected DNA cubes elicit RIG-I stimulation compared to hybrid RNA/DNA duplexes (Figure S6b) and all RNA cube assemblies trigger a significantly higher RIG-I stimulation than DNA cubes. Interestingly, the most immunostimulatory responses arise from RNA cubes produced from RNA/DNA fibers (Figure 3f). The overall viability of both reporter cell lines is comparable for all transfected samples without any significant difference from cells incubated with control reagents (Figure S5).

### Molecular Dynamics (MD) Simulations

**Figure 4:**
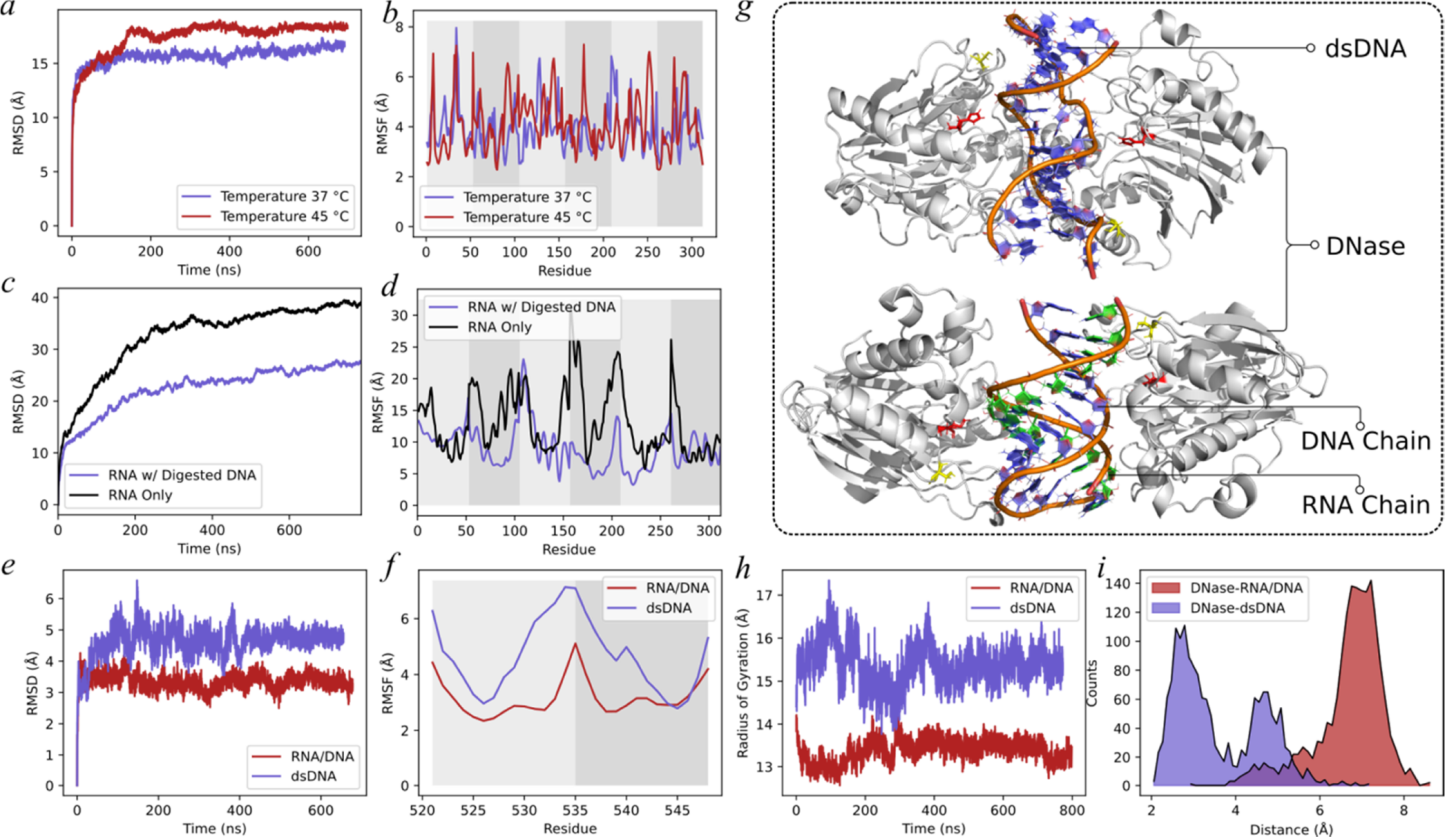
Molecular dynamics simulation results. (a, b) Root-mean-square deviation (RMSD) and root-mean-square fluctuation (RMSF) analyses of the RNA cube structure under 37 and 45 °C. (c, d) RMSD and RMSF analyses of the RNA-only structure and the RNA with the digested DNA structure. The striped shading of the background in panels (b) and (d) indicates different RNA chains. (e, f) RMSD and RMSF analyses of the DNase/RNA/DNA complex and the DNase/dsDNA complex. The striped shading of the background in panel (f) indicates different RNA and DNA chains. (g) Centroid structure of the largest cluster from the simulation of the DNase/RNA/DNA complex and the DNase/dsDNA complex. (h) Radius of gyration analysis of the two complexes. (i) Distribution of the distances between the active site (H134) in the DNase and the DNA chains.

We employ MD simulations to investigate the stability and dynamics of RNA structures at two different temperatures, 37 and 45 °C. Our findings (Figure 4a,b) indicate that the root-mean-square deviation (RMSD) and root-mean-square fluctuation (RMSF) values of the RNA cube at 45 °C were higher than those at 37 °C, suggesting that the thermostability of the cube structure is reduced at the higher temperature. To further explore the effects of DNA strand digestion on RNA structures, we conduct simulations for RNA-only and RNA with digested DNA structures—the RNA-only configuration comprised six RNA chains arranged at random. In contrast, the RNA/DNA hybrid encompasses multiple 5-mer DNA fragments that are complementary to the bases present in the 6 RNA chains. The RMSD and RMSF analyses (Figure 4c,d) reveal that the interaction between the RNA chains and digested DNA leads to an elevation in the thermostability of the RNA chains, hindering their ability to form the cube structure due to base pairing interactions between RNA and DNA nucleotides. Finally, we performed simulations for DNase with dsDNA and DNase with RNA/DNA duplexes. The RMSD and RMSF analyses (Figure 4e,f) reveal that the RNA/DNA complex is more stable than dsDNA upon binding the DNase. In addition, the radius of gyration of dsDNA (Figure 4h) is observed to be greater than that of the RNA/DNA complex. This observation suggests that the DNase exerts a more pronounced stretching effect (Figure 4g) on the dsDNA, which may have increased the likelihood of the DNase cleavage site encountering the DNA chains. Conversely, the RNA chains in the RNA/DNA complex impart a higher degree of rigidity compared to dsDNA. Notably, we compute the distances between the active site of the DNase (H134) and the DNA chains and observe that the distances in the DNase/dsDNA complex are significantly shorter than those in the RNA/DNA complex (Figure 4i).^23^ This finding elucidates the mechanism underlying the faster rate of DNase-mediated digestion of dsDNA compared to the RNA/DNA complex.

## Discussion

The nuclease-driven assembly of NANPs is based on degradation of the inert precursors made of either short RNA/DNA duplexes or longer RNA/DNA hybrid fibers. We show that the addition of either RQ1 DNase or RNase H affects the dynamics and yields of the subsequent assembly of NANPs. While the RNase H has evolved to efficiently recognize and cleave RNAs in RNA/DNA hybrid structures, the slower degradation of hybrids by DNase stems from its preference for the B-form helix, characteristic for dsDNA, rather than the A-form analogue, presented in RNA/DNA hybrids. Under these conditions, DNase residual activity is less than two percent^24^ and results in much slower release of the ssRNA strands. Furthermore, we speculate that the incubation temperature below the melting temperature of the RNA cube may allow misfolding and formation of undesired secondary structures with larger aggregates, which can also occur due to the lower rates of DNase digestion and RNA release. In addition, partially cleaved DNA strands that are still hybridized with their RNA counterparts may hinder the intended intermolecular interactions needed for the formation of RNA cubes.

An alternative approach for conditional assembly of NANPs is the application of strands that engage in toehold interactions with opposing strands, resulting in the branch migration and liberation of individual strands.^25−28^ However, this approach requires the presence of cognate NANPs, and the participating strands can interfere with the assembly process and alter the functionality of the products. Comparatively, in our method, all of the “masking” material is degraded, thus preventing any hampering in the assembly and function of NANPs.

Nuclease-driven assemblies were shown to have similar immunostimulatory properties to their counterparts that are prepared by a standard annealing protocol. While DNA cubes are not immunostimulatory, RNA cubes produced from digestion of RNA/DNA hybrids elicit an immune response similar to the control RNA cubes. Interestingly, RQ1 DNase-driven RNA cubes made from RNA/DNA fiber precursors induce the strongest responses, significantly higher than the one from control RNA cubes. The explanation of increased stimulation requires future investigation but may likely be caused by uncharacterized byproducts of partial DNA cleavage.

## Conclusions

In summary, we described a new one-step protocol for the conditional nuclease-driven assembly of either RNA or DNA cube NANPs with distinct immunostimulatory profiles. Nuclease-driven formation of NANPs only requires physiological temperature and the addition of substrate-specific nucleases. Together with the temperature stability of source materials, our approach allows storage and assembly independent of cold chain and complex laboratory equipment. We also suggest and explore a mode that might be a potential solution to enhance the stoichiometry of building materials, thus increasing the efficiency of full cube assembly. A possible way would be an arrangement of individual monomer strands on one scaffold. Although only a mono-directional approach of degradation (RNA monomers on DNA scaffold) was investigated, we believe that it could be engineered for bi-directional modes in the future. Indeed, complexing all monomers into one long RNA/DNA fibers functionally outperformed other constructs. Processing of simple nucleic acid-based therapeutics by cellular enzymes such as Dicer or RNA ligase has already been described to enhance the functionality of the delivered construct.^29,30^ Therefore, the further development of the “break to build” method, besides the streamlining of NANP assembly, may result in the design of nanoparticles that will assemble intracellularly by using cellular enzyme machinery.

## Material and Methods

### NANP Synthesis

All DNA templates and primers for PCRs and monomers for DNA cubes and fibers were purchased from IDT (the list of sequences is available in the SI). PCR was used to amplify RNA cube strand transcription templates using MyTaq Mix (Bioline). The PCR products were then purified by DNA Clean and Concentrator kit (Zymo Research). RNA cube monomers were transcribed by in vitro runoff transcription with T7 RNA polymerase in 80 mm HEPES-KOH (pH 7.5), 2.5 mm spermidine, 50 mM DTT, 25 mm MgCl2, and 25 mM each rNTP at 37 °C for 3.5 h. RQ1 RNase-free DNase (Promega) was used to degrade DNA templates before RNA purification by 8 M urea PAGE 8%AA (29:1). Excised bands were cut and overnight-eluted in crush and soak buffer (300 mm NaCl, 89 mm tris-borate [pH = 8.2], 2 mm EDTA). Eluted RNA strands were mixed with 100% EtOH in a 1:1.5 ratio and placed in −20 °C for at least 4 h. The mixture was centrifuged at 14,000 rcf for 30 min. Pellets were rinsed three times with 90% ethanol. Samples were dried by SpeedVac at 55 °C, and RNA pellets were resuspended in endotoxin-free water. Absorbances were measured using a NanoDrop 2000. NANPs were assembled at an equimolar ratio of each monomer. DNA or RNA monomers were mixed and denatured at 95 °C for 2 min, transferred to 45 °C, and incubated for 2 min; after that, assembly buffer (89 mm tris-borate [pH 8.2], 2 mm MgCl2, 50 mm KCl) was added, and solution was additionally incubated at 45 °C for 20 min. Six duplexes were individually prepared by the incubation of complementary RNA and DNA strands at 95 °C for 2 min, followed by the addition of the assembly buffer and incubation at room temperature for 20 min. RNA/DNA fibers were assembled by first assembling individual duplexes and then mixing them in an equimolar ratio to a single solution and allowing to assemble for 5 min at 37 °C. Assemblies were confirmed either on agarose gels or on an 8% (37.5:1) non-denaturing (native-PAGE) polyacrylamide gel with running buffer containing 89 mm tris-borate (pH 8.2) and 2 mm MgCl2. The gels were run in a Mini-Protean electrophoresis apparatus at 200V for 45 min and stained with ethidium bromide for 5 min.

### AFM

AFM imaging was carried out on a freshly cleaved mica surface submerged in an aqueous solution of APS (10(3-aminopropyl)-silatrane) by a MultiMode AFM Nanoscope IV system (Bruker Instruments) in tapping mode. Images were processed by the FemtoScan Online software package (Advanced Technologies Center, Moscow, Russia).

### Nuclease Treatment

The 5% (v/v) of either RQ1 DNase (Promega) or RNase H (Promega or New England Biolabs) were added to the mixture of six RNA/DNA hybrid duplexes based on the total volume of the sample, while 5% of ddiH2O was added instead of nuclease for control. Samples were incubated at 37 °C at different time points. Samples were aliquoted at selected time points and stored at 4 °C until ready for loading on gels.

### Kinetics

Quantification of band intensities was performed using ImageLab software version 6.0.1. Lanes and bands were manually defined. Kinetics were graphed using OriginPro 2023, and the sigmoidal fit was performed using a Boltzmann function.

### Stability Experiments

An IR vacuum concentrator (Labconco) was used to dry all samples at 55 °C and with IR radiation to obtain dehydrated duplexes, fibers, and cubes. Sample tubes (both dried and in solution) were placed on a heat block at 50 °C for 24 h. Samples were resuspended back to the original volumes using endotoxin-free water and analyzed by native-PAGE.

### Reporter Cell Assays

HEK-Blue hTLR7 and HEK-Lucia RIG-I cells, purchased from InvivoGen and maintained according to the supplier’s guidelines in an incubator at 37 °C and 5% CO_2_, are plated at 10,000 cells per well for RIG-I and 40,000 cells per well for HTLR7 in a 96-well plate 24 h prior to transfection of all samples using Lipofectamine 2000. 10 ng/mL 3p-hpRNA for HEK-Lucia RIG-I or 2 μg/mL R848 for HEK-Blue hTLR7 is used as a positive control for the indicated cell line. Addition of QUANTI-Blue solution to the HEK-Blue cell line or QUANTI-Luc solution to HEK-Lucia RIG-I cell line following the manufacturer’s protocol gave results for SEAP production or luciferase activity under the effect of 24 h treatment. A Tecan Spark plate reader was used to read the absorbance at 638 nm for QUANTI-Blue after 4 h of incubation at 37 °C. For QUANTI-Luc, the plate was read immediately for luminescence with a 100 ms reading time.

### Computational Simulations

The MD simulations were conducted using the pmemd.cuda program within the Amber 18 software package. We utilized the Amber RNA OL3 force field and protein ff14sb force field, which has been previously demonstrated to yield satisfactory results when studying the stability and dynamics of RNAs. The starting structures of RNA-only and RNA with digested DNAs were generated using the iFoldRNA program.^31−33^ The structure of the DNase I–DNA complex (PDB ID: 2DNJ) was used as a template to build the initial structure of DNase/dsDNA and the DNase/RNA/DNA complex. All of the structures were prepared using the teLeap module in Amber 18. The molecules were solvated in an octahedral box containing TIP3P water molecules, with a distance of 9Å maintained between atoms and the box boundary. We added potassium ions to neutralize the system, followed by additional K^+^ and Cl^–^ ions to achieve a concentration of approximately 0.3 M. We conducted several energy minimization steps before the actual simulation. First, we employed the steepest descent method for 1000 steps, followed by 1000 steps using the conjugate gradient method to perform energy minimization of the entire system. During the MD simulation phase, we initially applied weak restraints to the molecules and allowed the system to heat up from 0 K to the designated temperature (37 or 45 °C) over 200 ps. Afterward, we removed the restraints on the molecules and performed explicit solvent MD under constant pressure using the isothermal–isobaric (NPT) ensemble with a time step of 2 fs. The length of hydrogen bonds was constrained using the shake algorithm, while the temperature was maintained, and pressure was kept constant at 1 bar throughout the simulation.

## Supporting Information Available

DNA and RNA sequences used in the study; kinetics of the DNA cube RNase-driven assembly; kinetics of the RNase-driven DNA cube assembly; kinetics of the RNA cube DNase-driven assembly; effect of storage on duplex and cube assembly’s stability; cell viability of reporter HEK-Blue hTLR7 and HEK-Lucia RIG-I reporter cell line by transfected cubes, fibers, and duplexes; immunostimulatory properties of RNA or DNA cubes in reporter HEK-Blue hTLR7 and HEK-Lucia RIG-I reporter cells (PDF)
