# Supplementary material for: Break to Build: Isothermal Assembly of Nucleic Acid Nanoparticles (NANPs) via Enzymatic Degradation
Source: Bioconjug Chem. 2023 Jun 9;34(6):1139–46. doi: 10.1021/acs.bioconjchem.3c00167 (PMC10288440; doi:10.1021/acs.bioconjchem.3c00167)
Supplement: Supplementary file 1 — bc3c00167_si_001.pdf [file bc3c00167_si_001.pdf]

Supporting Information

Break to build: isothermal assembly of nucleic acid nanoparticles (NANPs) *via* enzymatic degradation.

Damian Beasock[†#], Anh Ha[†#], Justin Halman[†], Martin Panigaj[†], Jian Wang[‡], Nikolay V. Dokholyan[‡,§], Kirill A. Afonin[†*]

[†] Nanoscale Science Program, Department of Chemistry, University of North Carolina at Charlotte, Charlotte, NC, 28223, USA

[‡] Department of Pharmacology, Department of Biochemistry & Molecular Biology, Penn State College of Medicine, Hershey, PA, 17033, USA

[§] Department of Biochemistry & Molecular Biology, Department of Biochemistry & Molecular Biology, Penn State College of Medicine, Hershey, PA, 17033, USA

# - these authors contributed equally to this project

* To whom correspondence should be addressed. Tel: +1 704 687 0685; Fax: +1 704 687 0960; Email: kafonin@uncc.edu

SEQUENCES USED IN THIS PROJECT

RNA cube:

rA- 5’- GGCAACUUUGAUCCCUCGGUUUAGCGCCGGCCUUUUCUCCCACACUUUCACG

rB- 5’- GGGAAAUUUCGUGGUAGGUUUUGUUGCCCGUGUUUCUACGAUUACUUUGGUC

rC- 5’- GGACAUUUUCGAGACAGCAUUUUUUCCCGACCUUUGCGGAUUGUAUUUUAGG

rD- 5’- GGCGCUUUUGACCUUCUGCUUUAUGUCCCCUAUUUCUUAAUGACUUUUGGCC

rE- 5’- GGGAGAUUUAGUCAUUAAGUUUUACAAUCCGCUUUGUAAUCGUAGUUUGUGU

rF- 5’- GGGAUCUUUACCUACCACGUUUUGCUGUCUCGUUUGCAGAAGGUCUUUCCGA

rD-Al488:

5'- GGCGCUUUUGACCUUCUGCUUUAUGUCCCCUAUUUCUUAAUGACUUUUGGCC/Al488/

DNA cubes with three As at each corner (all strands are complementary to corresponding cube strands):

anti-dA- 5’- CGTGAAAGTGTGGGAGAAAAGGCCGGCGCTAAACCGAGGGATCAAAGTTGCC

anti-dB- 5’- GACCAAAGTAATCGTAGAAACACGGGCAACAAAACCTACCACGAAATTTCCC

anti-dC- 5’- CCTAAAATACAATCCGCAAAGGTCGGGAAAAAATGCTGTCTCGAAAATGTCC

anti-dD- 5’- GGCCAAAAGTCATTAAGAAATAGGGGACATAAAGCAGAAGGTCAAAAGCGCC

anti-dE- 5’- ACACAAACTACGATTACAAAGCGGATTGTAAAACTTAATGACTAAATCTCCC

anti-dF- 5’- TCGGAAAGACCTTCTGCAAACGAGACAGCAAAACGTGGTAGGTAAAGATCCC

anti-dDAl546:

5’- /Al546/GGCCAAAAGTCATTAAGAAATAGGGGACATAAAGCAGAAGGTCAAAAGCGCC

DNA strands required to form RNA/DNA fibers when mixed with RNA cube strands

For the RNA/DNA fibers, the 15mer overlaps have been color-coded to reflect their intended interactions.

Holding A
5'TAGGCACCTTGCTAACGTGAAAGTGTGGGAGAAAAGGCCGGCGCTAAACCGAGGGATCAAAGTTGCC

Holding B
5'TTAGCAAGGTGCCTAGACCAAAGTAATCGTAGAAACACGGGCAACAAAACCTACCACGAAATTTCCCGTATACGACGCGCTA

Holding C
5'CTACGGTCATATTGCCCTAAAATACAATCCGCAAAGGTCGGGAAAAAATGCTGTCTCGAA
AATGTCCTAGCGCGTCGTATAC

Holding D
5'GCAATATGACCGTAGGGCCAAAAGTCATTAAGAAATAGGGGACATAAAGCAGAAGGTCAA
AAGCGCCGTCACTGATGTCAGA

Holding E
5'ACGACTGTCAGGTCAACACAAACTACGATTACAAAGCGGATTGTAAAACTTAATGACTAAA
TCTCCCTCTGACATCAGTGAC

Holding F
5'TGACCTGACAGTCGTTCGGAAAGACCTTCTGCAAACGAGACAGCAAAACGTGGTAGGTA
AAGATCCC

SUPPORTING FIGURES

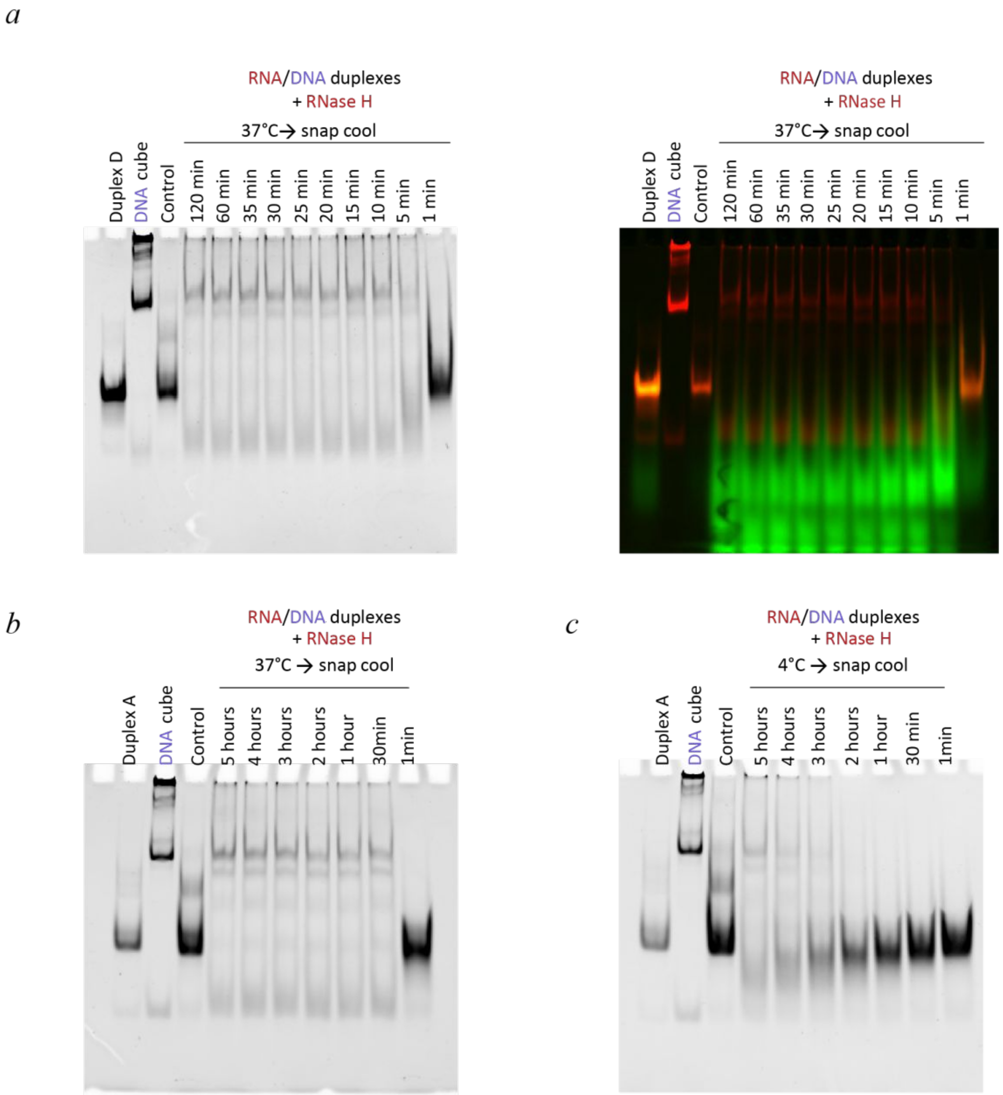

Supporting Figure S1: Kinetics of RNase H-driven DNA cube assembly assessed at different temperatures. (a) Fluorescently labeled (with Alexa 488 and Alexa 546) RNA/DNA hybrid duplexes were treated with RNase H and incubated at either 37°C or 4°C from 1 minute to 2 hours. The reactions were stopped by snap cooling in liquid nitrogen. (b, c) RNA/DNA hybrid duplexes treated with RNase H incubated at either 37°C or 4°C from 1 minute to 5 hours. Control samples were treated with endotoxin free water instead of RNase H and incubated for 5 hours at either 37°C or 4°C. Interestingly, the second band is visible in nuclease-driven assembly but not in control DNA cubes. The likely explanation is that the two bands appear as an artifact arising from snap freezing in liquid nitrogen that caused cold-denaturation leading to destabilization of the DNA cube structure.

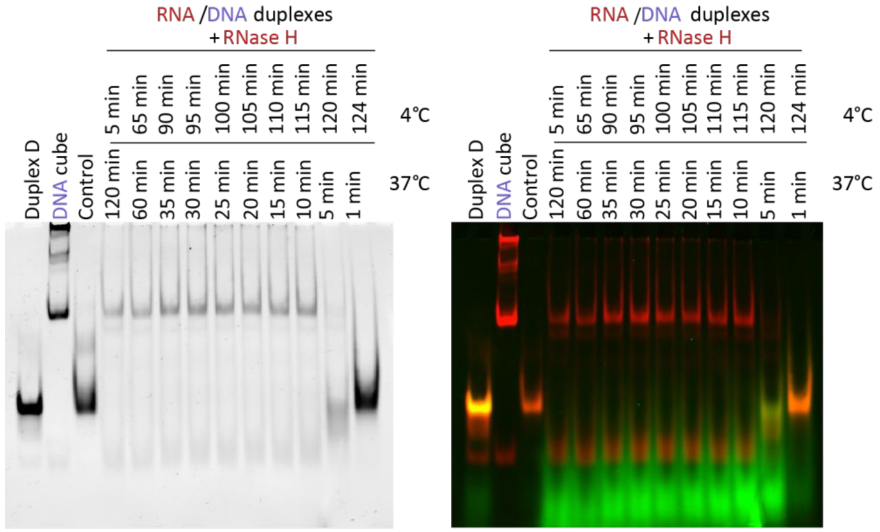

Supporting Figure S2: Kinetics of RNase H-driven DNA cube assembly at 37° C followed by incubation at 4°C. RNA/DNA hybrid duplexes fluorescently labeled with Alexa 488 and Alexa 546 were treated with RNase H from 1 minute to 120 minutes at 37° C and stored at 4°C.

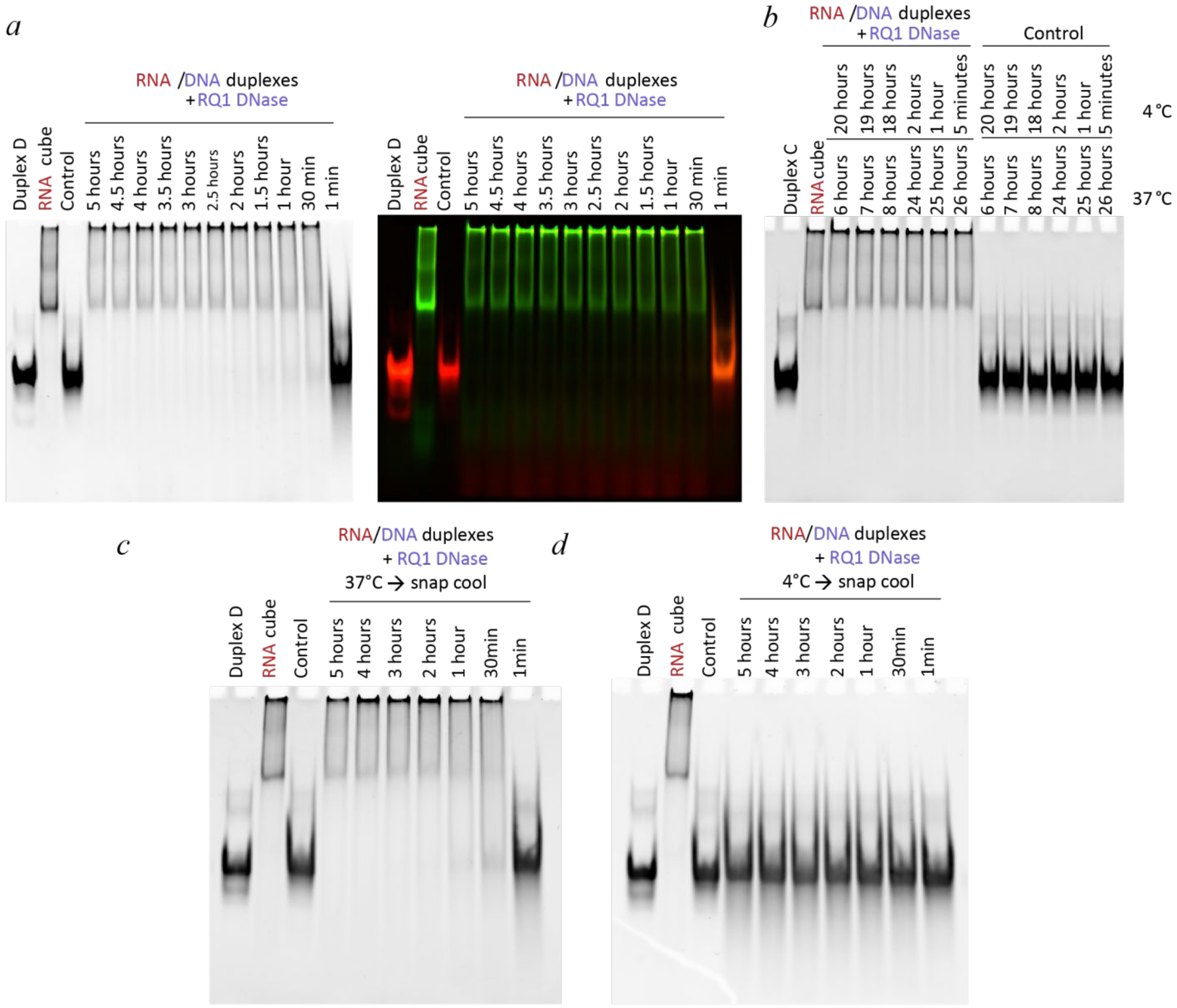

Supporting Figure S3: Kinetics of RQ1 DNase-driven RNA cube assembly. (a) Fluorescently labeled (with Alexa 488 and Alexa 546) RNA/DNA hybrid duplexes were treated with RQ1 DNase from 1 minute to 5 hours at 37 °C. (b) RNA/DNA hybrid duplexes treated with RQ1 DNase were incubated at 37°C from 6 hours to 26 hours and stored at 4°C for indicated time. Control samples were treated with endotoxin free water instead of RQ1 DNase and incubated 26 hours at 37°C and stored at 4°C for 5 minutes. (c, d) RNA/DNA hybrid duplexes treated with RQ1 DNase incubated at 37°C or 4°C from 1 minute to 5 hours; the reactions were stopped by snapped cooling in liquid nitrogen. Control samples were treated with endotoxin free water instead of RQ1 DNase and incubated 5 hours at either 37°C or 4°C.

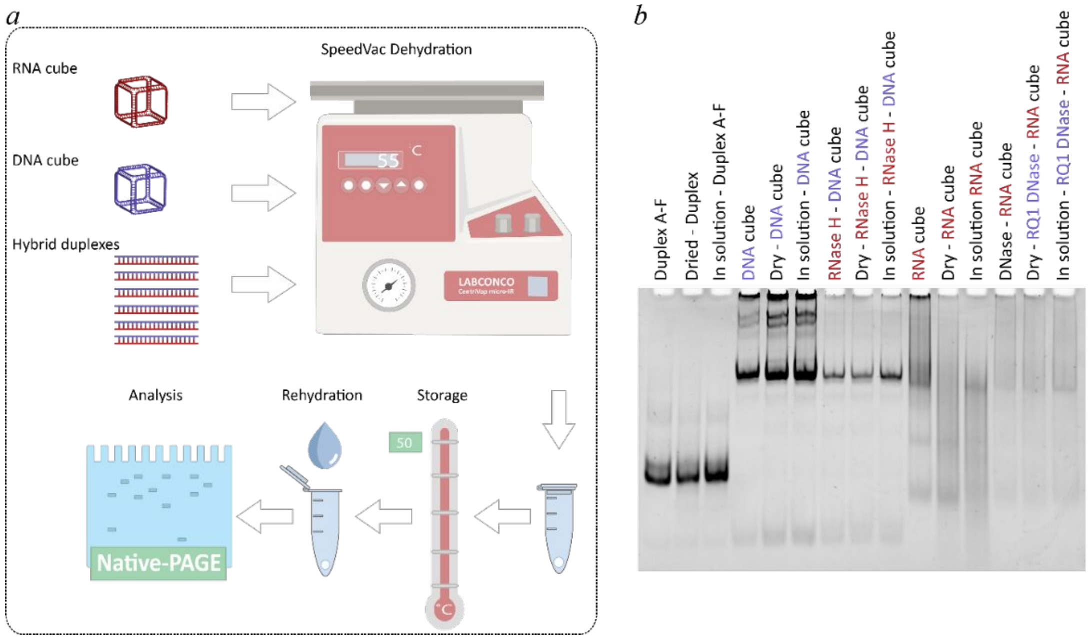

Supporting Figure S4: Effect of storage conditions on stabilities of RNA/DNA hybrid duplexes, controlled cubes, and nuclease-driven cube assemblies. (a) Schematic of experimental workflow for RNA cubes, DNA cubes and RNA/DNA hybrid duplexes storage under elevated temperature. (b) Native-PAGE analysis of structural integrities of DNA cubes, RNA cubes, and RNA/DNA hybrid duplexes after storage at +50°C either in solution or in dehydrated state.

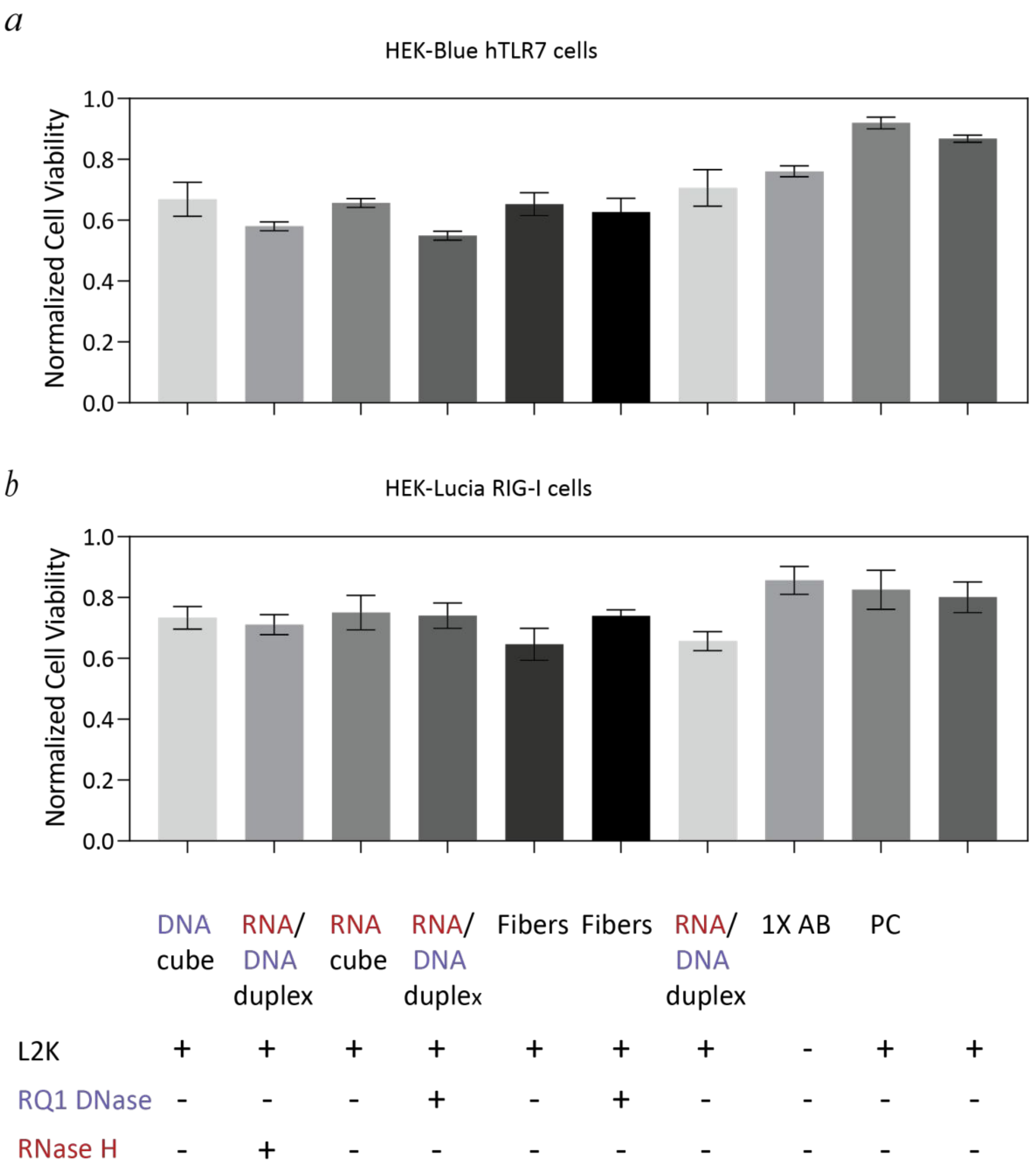

Supporting Figure S5: Cell viability of reporter HEK-Blue hTLR7 and HEK-Lucia RIG-I reporter cell line by transfected cubes, RNA/DNA fibers, and RNA/DNA duplexes. (a) Normalized cell viability in treated HEK-Blue hTLR7 cell line. n= 4, ±SEM. (b) HEK-Lucia RIG-I viability, n= 3, ±SEM. Normalized to untreated cells.

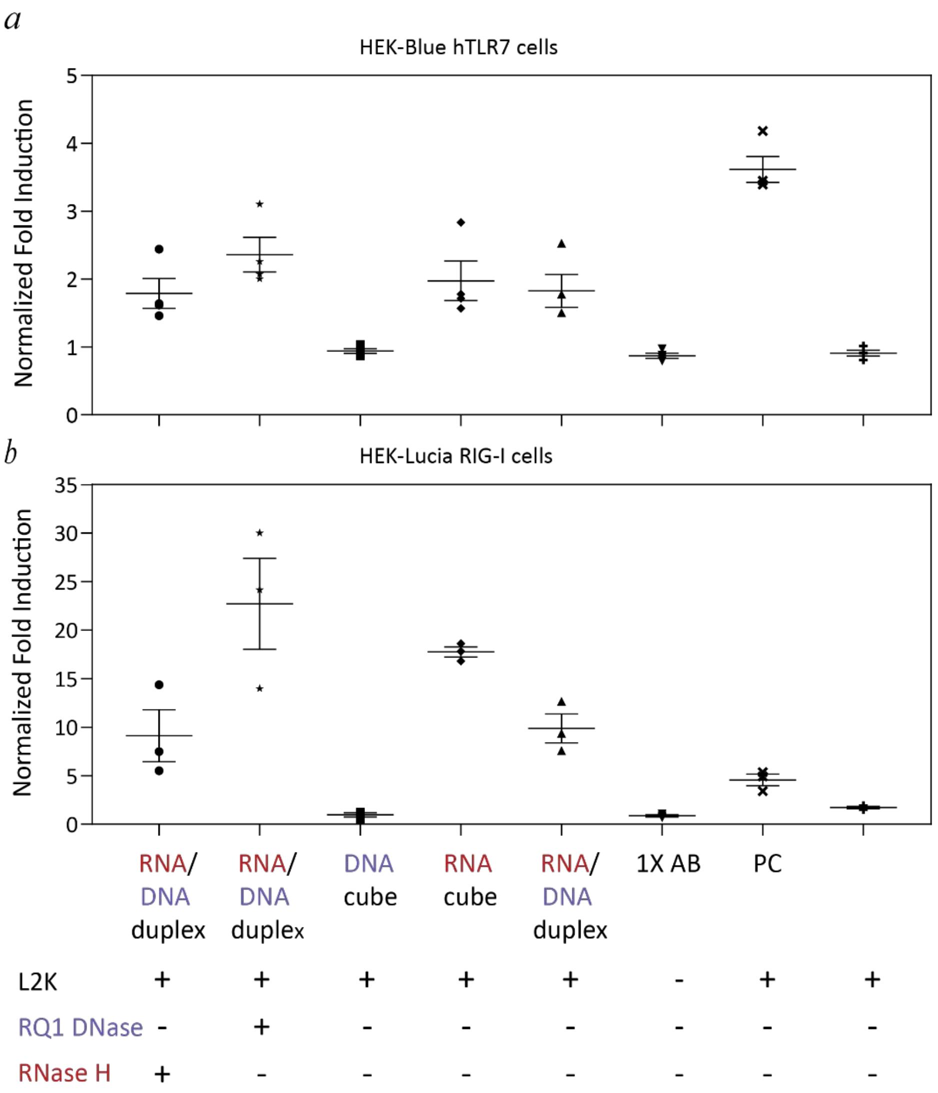

Supporting Figure S6: Immunostimulatory properties of RNA or DNA cubes in reporter HEK-Blue hTLR7 and HEK-Lucia RIG-I reporter cells. Normalized to untreated cells.
